# High GLI-1 Expression is a Reliable Indicator of Bad Prognosis in Newly Diagnosed Acute Leukemia Patients

**DOI:** 10.1007/s12288-022-01609-y

**Published:** 2023-01-03

**Authors:** Reham S. El Zaiat, Reem Nabil, Khaled A. Khalifa, Aliaa A. El Feshawy

**Affiliations:** 1 Faculty of Medicine, Clinical Pathology Department, Menoufia University, Shebein El kom, Egypt; 2 Clinical Pathology Department, National Cancer Institute, Cairo University, Cairo, Egypt

**Keywords:** GLI-1, Acute myeloid leukemia, Hedgehog signaling, FLT3, APL

## Abstract

Purpose: To explore the expression and prognostic significance of Hedgehog signaling transcription factor GLI-1 in newly diagnosed acute myeloid leukemia (AML) patients. Methods: Clinical specimens were obtained from 46 recently diagnosed AML patients. Real-time qPCR was used to measure the GLI-1 mRNA expression in bone marrow mononuclear cells.Also, the relationship between GLI-1 mRNA levels and clinical variables and prognostic variables was assessed. Results: GLI-1 was overexpressed in the bone marrow samples of our patients. GLI-1mRNA expression did not differ significantly across different age groups, between both sexes, or between different FAB subtypes (P = 0.882, P = 0.246, and P = 0.890, respectively). GLI-1 expression varied significantly in different risk categories, with the greatest levels observed in 11 patients with poor risk (24.6 versus 22.7) compared to intermediate risk (5.2 versus 3.9; P = 0.006) and favorable risk (4.2 versus 3; P = 0.001). Comparing patients with the wild FLT3 allele to those with the mutant one, GLI-1 gene levels were considerably greater in those with the mutant allele of FLT3.Following induction chemotherapy, the levels of GLI-1 mRNA were significantly higher in 22 patients who did not experience complete remission (CR) diagnosed with de novo non-acute promyelocytic leukemia (APL) compared to 17 patients who did (P = 0.017). Significantly greater levels of expression were observed in each category of the patients with favorable risk; wild FLT3 allele (P = 0.033) and CR failure P = 0.005). Conclusion: GLI-1 overexpression is a risk factor for poor prognosis and could be a novel therapeutic target for AML.

## Introduction

AML is a complex disease characterized by the deregulation of numerous signaling pathways that lead to wide molecular alterations. These signaling pathways can be considered as potential therapeutic targets in AML treatment strategies [1].

One of these crucial routes is the Hedgehog (Hh) signaling pathway, a key pathway in stem cell and embryonic development, It was discovered by Christiane Nusslein-Volhard and Eric F. Weischaus in 1980 [1, 2].

The Hh pathway is fairly active in adult tissues and it can be stimulated under certain circumstances such as during wound healing [3]. Moreover, the system plays a role in the maintenance of somatic stem cells and pluripotent cells necessary for tissue repair [4].

According to the most recent estimates, it plays a role in the development of one-third of all malignant tumors when it is aberrantly activated by activating GLI transcription factors; which are the primary downstream effectors of the Hh signaling cascade [5].

Additionally, its significance in hematological malignancies like leukemias, B-cell lymphomas, and multiple myelomas has been established [6].

The location where Hh ligands bind to initiate Hh signaling is the transmembrane receptor Patched 1 (PTCH1), which derepresses the transmembrane G protein-coupled receptor Smoothened (SMO). As a result, the terminal effectors of the Hh pathway, the glioma-associated oncogene homolog 1 (GLI-1) transcription factor, are broken from the suppressor of fused (SUFU)-mediated cytoplasmic sequestration, allowing nuclear translocation and activation of target genes [1].

Mammals have three GLI isoforms: GLI-1 which increases gene expression and is a trustable biomarker of pathway stimulation, GLI-2 which can either repress or induce gene expression, and GLI-3 which works as a transcriptional repressor [7].

Although GLI-1 is not highly expressed in differentiated tissues, abnormal activation of the protein has been linked to the promotion of several hallmarks of cancer, including proliferation, angiogenesis, metastasis, survival, metabolic renewal, and resistance to chemotherapy. These actions are motivated by the potential function of GLI-1 in cell cycle control [1].

According to Wellbrock and colleagues’ observations, an increased serum level of Desert Hedgehog (DHH) was seen in AML patients during its into the blood by the bone marrow microenvironment rather than the AML cells [8]. Others, report higher levels of Hh effectors in post-MDS AML and increased expression of Sonic Hedgehog (SHH), SMO, and GLI-1 in BM stromal cells of MDS patients. More recently, it has been demonstrated that high-risk MDS and elevated DNMT1 expression are correlated with GLI-1 expression [9].

Association between high GLI-1 expression, poor prognosis [8], and chemotherapeutic resistance in AML [5] were also reported.

Therefore, we conducted this study to explore more about the predictive importance of GLI-1 transcription factor expression in AML patients.

## Methods

The study included 46 newly diagnosed AML patients, recruited from Hemato-Oncology and Clinical Pathology Departments, National Cancer Institute, Cairo University, Egypt in the period from October 2019 to January 2021.The 7 patients diagnosed with acute promyelocytic leukemia (APL) were excluded from the statistical analysis as regards the disease outcome .

The study was approved by Ethical committee of Menoufia faculty of medicine (19,819 CPATH) and informed consent was taken from each participant.

Their medical records were reviewed for reporting clinicopathological and survival data.

AML diagnosis was based on full clinical assessment and routine laboratory work in the form of peripheral blood evaluation, bone marrow examination, cytochemistry, and immunophenotyping. For risk stratification: cytogenetic analysis was done by karyotyping using IKAROS imaging system (Metasystems, Altlussheim, Germany) and molecular study of FLT3 mutation were detected by conventional PCR using Applied Biosystems 3500 Genetic Analyzer (Applied Biosystems, CA, USA). DNA extraction and the domain of FLT-3 was PCR amplified and products were analyzed and the amplicons with a size greater than that of wild type (328 ± 1 bases) were interpreted as positive for the ITD mutation.

Detection of expression levels of GLI-1 gene in AML bone marrow samples was done by 7460 Real-Time PCR System (Applied Biosystems, CA, USA). The normal calibrator of our results was the mean level of GLI-1 expression in additional morphological normal bone marrow samples.

RNA was extracted from bone marrow mononuclear cells collected on EDTA using QIAamp RNA Blood Mini Kit for total RNA purification (QIAGEN, Hilden, Germany). The purity of the isolated total RNA were evaluated using a NanoDrop (2000 spectrophotometer, Thermo Scientific).The absorbance at 260 and 280 nm (A260/280) ratio was employed. A260/280 for pure RNA is 2.The High-Capacity cDNA Reverse Transcription Kit from Applied Biosystems was used for reverse transcription; (Thermo Fisher Scientific, USA; catalogue no. 4,387,406). The cDNA was then kept for future use at -20 °C.

GLI-1 expression quantification by Real-time PCR was carried out using TaqMan® Universal PCR Master Mix (Catalog no.: 4,370,048, Thermo FisherScientific, Applied Biosystems, USA) Primers` sequences were designed for GLI-1 gene as : F 5’- CCACGGGGAGCGGAAGGAG, R 5’- ACTGGCATTGCTGAAGGCTTTACTG and for GAPDH (House keeping gene) as F 5’- TACACTGAATTCACCCCCAC-3’, R 5’- CATCCCAATCCAAATGCGGCA − 3’.

## Results Interpretation

The comparative Ct (ΔΔCt) method was used to determine the relative target quantity in samples (ΔΔCt = ΔCt case -ΔCt control).With the comparative Ct method, the 7460 software measures the amplification of the target and the endogenous control in patients’ samples and reference samples. Measurements were normalized using the endogenous control (ΔCt case/control = Ct GLI-1- Ct GAPDH). GLI-1 expression was expressed as a fold change of control.

The response was assessed at D28 post-induction chemotherapy, patients were considered to have complete remission (CR) if marrowcellularity of ≥ 20%, the neutrophil count was ≥ 1 × 10^9^ and marrow blasts were less than 5%. While those who failed to achieve those levels were considered as a non-CR group.

Patients were defined to have a favorable, intermediate, or poor risk based on the cytogenetic results and FLT3mutation status. The cytogenetic risk was determined according to the 2016 WHO classification. Patients with intermediate-risk cytogenetics were considered to have intermediate-risk if they harbor normal FLT3; however, they were assigned to the poor-risk group if they tested positive for FLT3 mutation [10].

### Statistical Analysis

Analyzed using IBM SPSS software package version 20.0. (Armonk, NY: IBM Corp). Qualitative data were described using number and percent. Quantitative data were described using range, mean, median and interquartile range. Significance of the obtained results was judged at the 5% level. The Mann–Whitney test was used for abnormally quantitative variables, to compare between two studied groups. The Kruskal–Wallis test was used to compare more than two groups. Receiver operating characteristic curve (ROC) was used to plot for GLI-1 expression; the area under the ROC curve denotes the performance and the 95% confidence intervals. Overall survival distributions were plotted using Kaplan–Meier estimates. **Results**:

Table 1 shows demographic and clinical data of the patients .

**Table 1:** Demographic, Clinical, and survival status of the patients (n = 46)

	No.	%
**Age (years)**		
18– 40	22	47.8
40–60	14	30.4
> 60	10	21.7
Min. – Max.	18.0–90.0	
Mean + SD.	45.0 ± 17.48	
Median(IQR)	44.0(31–56)	
**Gender**		
Male	27	58.7
Female	19	41.3
**Organomegaly**		
Splenomegaly	43	93.5
Hepatomegaly	23	46.0
Hepatosplenomegaly	20	43.5
Lymphadenopathy	28	60.9
**FLT3-ITD**		
Wild	28	60.9
Mutant	18	39.1
**Risk stratification**		
Favorable	21	45.7
Intermediate	14	30.4
Poor	11	23.9
**Response at day 28**		
Achieved CR	22	47.8
Not achieved CR	24	52.2
**Survival status**		
Dead	20	43.5
Alive	26	56.5

Distribution of FAB subtypes in the patients’ group demonstrated that 7 patients were diagnosed as APL (15.2%) and the remainder (39 patients) were non-APLAML patients (84.8%). Overall survival mean of our patients was 13.030 Months.

**Fig. 1:**
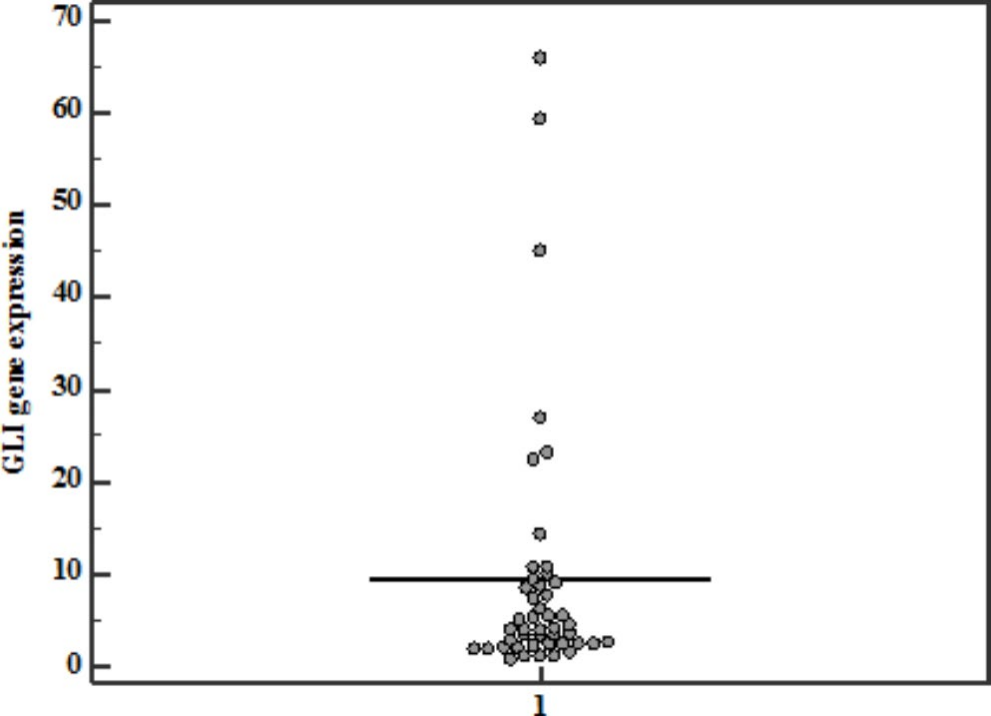
Descriptive analysis of the studied group according to GLI-1 gene expression (n= 46)

GLI-1 gene expression level among patients was ranging between (0.68–65.8) with a mean ± SD of 9.4 ± 14 (Fig. 1).

Study of GLI-1 gene expression in relation to patients’ characteristics revealed that no statistically significant difference was demonstrated between GLI-1 gene expression and different age groups, between males and females, or among different FAB subtypes (p = 0.175, 0.645, and 0.271, respectively), also, the expression wasn’t significantly different on comparing APL patients with other FAB subtypes (p = 0.661).

According to Table 2, there was significant difference in GLI-1 level between the various risk categories, with the poor risk group having the highest level (p = 0.002). Further analysis found that the poor risk group’s GLI-1 expression was significantly higher than that of the favorable and intermediate-risk groups (p = 0.001, p = 0.006)While there was no discernible difference between the favorable and intermediate-risk groups (p = 0.620). Patients harboring mutant FLT3 had significantly higher GLI-1gene expression level compared to those carrying the wild gene (p = 0.006).

**Table 2:** Relation between GLI-1 gene expression and risk (n = 46)

GLI-1 gene expression	Risk			H	p
	**Poor (n = 11)**	**Intermediate (n = 14)**	**Favorable (n = 21)**		
Min. – Max.	2.5–65.8	1.1–14.2	0.7–10.7	12.696^*^	0.002^*^
Mean ± SD.	24.6 ± 22.7	5.2 ± 3.9	4.2 ± 3		
Median	22.3	3.7	2.77		
**Sig.bet.Grps**	p_1_ = 0.001^*,^ p_2_ = 0.006^*^,p_3_ = 0.620				

p: p-value for comparing among the studied groups

p_1_: p-value for comparing between Poor and Favorable

p_2_: p-value for comparing between Poor and Intermediate

p_3_: p-value for comparing between Favorable and Intermediate

Investigating GLI-1 gene expression in relation to disease outcome in terms of patient’s response on day 28 (CR) and survival analysis was carried out on the 39 non-APL patients and revealed that GLI-1 was increased in those who did not achieve CR than those who were in CR after initial chemotherapy (p = 0.017).

The overall survival was significantly shortened in those who did not achieve CR than in those who were in CR after initial chemotherapy (p < 0.001). Further analysis of GLI-1 expression in relation tothe response within each risk subgroup showed that, there was no difference in the non-CR group compared to the CR group in both poor and intermediate risk. However, significantly higher levels were demonstrated in the non-CR group compared to the CR in favourable one (mean ± SD 3.6–10.7vs 0.7–10.7, p = 0.005) .

The relation of GLI-1 expression and response in the subgroups of wild and mutant FLT3, alive and dead patients was further assessed showing that significantly higher levels of that significantly higher levels of that gene were seen in the non-responders compared to the responders in both subgroups of wild FLT3 subgroup and alive patients.While, in the mutant group or those who did not survive, there was no difference between the CR and non-CR **(**Table 3**)**.

**Table 3:** GLI gene expression in relation to response within each risk subgroupwild versus mutant FLT3-ITD and alive versus dead patients

	N	GLI-1 gene expression			U	P
		**Min.-Max.**	**Mean ± SD.**	**Median**		
**Risk stratification** **Favorable** No CR CR	6 15	3.6–10.7 0.7–10.7	6.7 ± 3.1 3.1 ± 2.4	5.9 2.5	10.50^*^	0.005^*^
**Intermediate** No CR CR	**9** **5**	1.1–14.2 1.6–9.5	5.2 ± 4.3 5.3 ± 3.5	4.0 3.4	19.0	0.699
**Poor** No CR CR	**9** **2**	2.5–65.8 8.9–59.3	22.5 ± 21.3 34.1 ± 35.6	22.3 34.1	6.0	0.582
**FLT3-ITD** **Wild** No CR CR	**12** **16**	1.8–10.0 0.7–10.7	5.2 ± 2.6 3.5 ± 3.0	4.3 2.4	50.50^*^	0.033^*^
**Mutant** No CR CR	**12** **6**	1.1–65.8 3.3–59.3	18.2 ± 20.1 15.5 ± 21.6	10.2 8.8	35.0	0.964
**Alive** No CR CR	**7** **19**	3.3–44.9 0.7–59.3	18.0 ± 13.9 7.5 ± 13.0	14.2 3.4	24.0^*^	0.013^*^
**Dead** No CR CR	**17** **3**	1.1 – 65.8 1.9–2.5	9.1 ± 15.7 2.2 ± 0.3	4.4 2.3	9.50	0.093

U: Mann Whitney test, p: p-value for comparing different parameters

*: Statistically significant at p ≤ 0.05

**Fig. 2:**
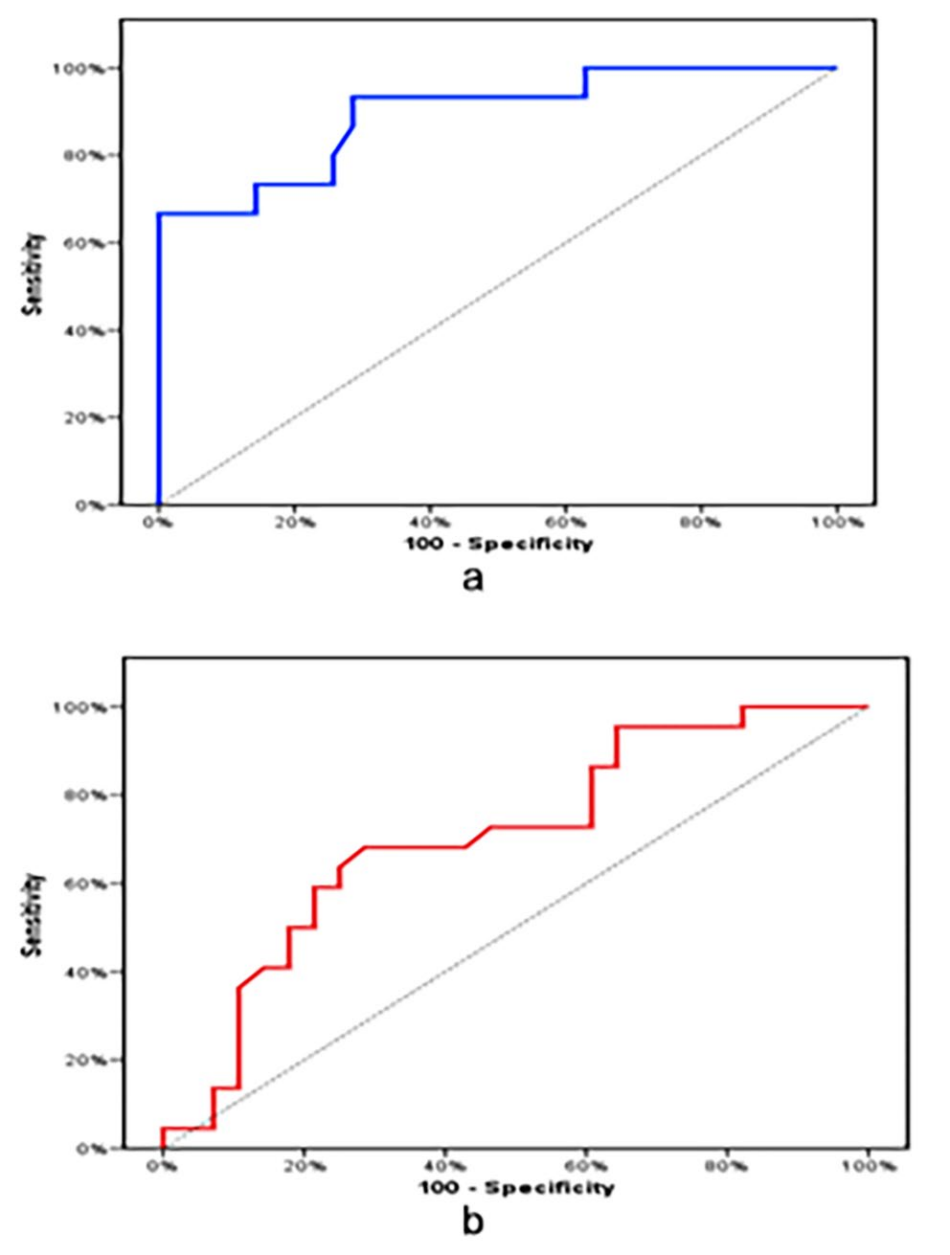
a. ROC curve for GLI-1 gene expression to predict poor risk b. ROC curve for GLI-1 gene expression to predict response at day 28

ROC curve showed that GLI-1 at cutoff level (> 5.1) can significantly predict patients developing poor risk with a sensitivity of (90.9%), specificity of (71.43%), a negative predictive value of (96.2%), and a positive predictive value of (50.0%) While GLI-1 at cutoff level (≤ 4) can detect day 28 chemotherapeutic response with a sensitivity of (68.18%), specificity of (66.67%),negative predictive value of (65.2%), and a positive predictive value of (69.6%) **(**Fig. 2**).**

## Discussion

In the present study, we aimed to evaluate GLI-1 gene expression level in newly diagnosed AML patients and to assess its prognostic role .

Our study included 46 newly diagnosed AML patients with slight male predominance (1.5 male: 1 female).Most of our patients (52%) were between 40 and 90 years old. This comes in agreement with the known data that AML is generally a disease of older people and is uncommon before the age of 45 [11].

The survival of AML patients and treatment response vary among different populations as they are determined by several factors including the patient`s age and the disease`s biological characteristics with very poor overall survival outcomes in older adults.

In our study, we revealed that GLI-1 was over-expressed in the patients’samples. GLI-1 overexpression is thought to be the secret beyond the role of Hh pathway dysregulation in tumorigenesis. It transcriptionally controls target genes implicated in cell proliferation, differentiation, and survival. So, overproduction of that gene has an oncogenic role in hematological and non-hematological tumors. **Zhou et al.**, emphasized that there was a transcriptional overexpression of GLI-1 in AML cell lines. They proposed that it promotes cell proliferation and cell cycle progression along with reducing chemotherapy sensitivity in AML cells. Blocking GLI-1 function in AML cells by using GLI**-**1antagonist (GANT-61) was investigated by **Lau et al.**, who demonstrated decreased proliferation, growth arrest, and apoptosis in AML cell lines [12, 13]. In CML, **Abraham et al.**, found that GLI-1 expression increased significantly upon disease progression from chronic phase to accelerated or to blastic crisis [14, 15]. In addition, the Hh pathway appears to have a potential role in leukemia stem cell (LSC) maintenance in CML relapse following therapy termination, according to **Lainez-G et al.**, [16].

In MDS, there was a link between Hh/GLI-1 activation and leukemic transformation. HH protein expression was higher in MDS cells than in non-MDS cells [17].

Compared to normal B cells, the elevated activity of MEK/RSK signaling in multiple myeloma cells is correlated with aberrant HH-GLI signaling. Combining the RSK inhibitor and the GLI inhibitor has a synergistic effect on lowering myeloma cell survival and activating the HH-GLI pathway.

GLI-1 had been demonstrated to be overexpressed in multiple human cancers including glioblastoma, osteosarcoma, and rhabdomyosarcoma. In gastrointestinal cancers and pancreatic cancers driven by KRAS/BRAF mutation, GLI-1 is over-activated. Recently, it has been proposed that oncogenic GLI-1 progresses through colon carcinogenesis and distant metastasis [18].

Overexpression of GLI-1 was significantly related to adverse prognostic markers in our patients as higher expression levels were seen in patients harboring mutant FLT3 allele who did not achieve CR than those who were in CR at D28.

The relation of the gene to FLT3 mutational status as one of the most important poor prognostic factors in AML was observed also by **Latuske et al.**, who found that overexpression of GLI-1 confers an adverse prognosis to AML patients as it was positively correlated with a mutated FLT3 [19]. **Wellbrock et al.**., also found that the effect of GLI expression on the AML patients’ survival was correlated to the occurrence of FLT3 mutational status [8].

Previous results can be explained by the possible crosstalk between the FLT3 pathway, HH pathway effector, and GLI-1 in AML cells, especially in patients with FLT3 activating mutations. This hypothesis was proven by examining the inhibition of FLT3 signaling in the FLT3-mutated AML cell lines, which had a direct impact on GLI protein expression in these cells. Combining the suppression of GLI, FLT3, and PI3K, in vitro,demonstrated potent anti-leukemic effects in FLT3-mutated AML cells compared to FLT3 wild-type AML cells [20].

Additionally, we observed significantly higher expression levels in the poor risk group compared to the favorable and intermediate risk groups while an insignificant difference was detected between the favorable and intermediate risk ones.

Patients^’^ cytogenetic category was and still a key determinant of AML prognosis and its relation to GLI-1 expression was investigated by **Terao&Minami**. They found a high GLI-1 expression in a poor prognosis as it correlated with poor cytogenetic risk, reduced overall survival, and can be a poor predictor of remission status [17].

We found that GLI-1 gene overexpression can only exhibit an adverse outcome and affect the achievement of initial response in patients who were in the favorable risk category or harbored wild FLT3allele.While it did not influence the initial response in the intermediate and poor risk patients or those who carried the mutant allele. This could be because the GLI gene was significantly overexpressed in the non-responders compared to responders in the favorable risk subgroup, and the wild FLT3 subgroup yet there was no statistical significance difference in non-responders compared to responders in both poor and intermediate risk or the subgroup of mutant FLT3.

To summarize, this research focuses on using GLI-1 as a true prognostic tool in real AML patients as we noticed that most of the recent and previous experiments aimed at providing an additional treatment option through monitoring the effect of GLI-1 inhibitors on AML cell lines without developing a real technique to identify those patients who would truly benefit from this treatment option [21–25].

The present study demonstrates that high expression of GLI-1 exhibits a poor risk feature in newly diagnosed AML patients, and it can be utilized in risk stratification remodeling and as a diagnostic predictor of the initial chemotherapeutic response especially in those who appear to have a favorable outcome.

## Conclusion

We believe that high GLI-1 expression could be used to identify a high-risk group of AML patients and assist clinicians in determining the patients who would benefit from adding GLI-1 inhibitors to their chemotherapy regimens.
